# Poor communication between ER and mitochondria: a signature of ALS/FTD?

**DOI:** 10.18632/aging.205206

**Published:** 2023-10-20

**Authors:** Christopher C.J. Miller, Patricia Gomez-Suaga

**Affiliations:** 1 Department of Basic and Clinical Neuroscience, Institute of Psychiatry, Psychology and Neuroscience, London, United Kingdom; 2 Universidad de Extremadura, Departamento de Bioquímica y Biología Molecular y Genética, Facultad de Enfermería y Terapia Ocupacional, Cáceres 10003, Spain; 3 Centro de Investigación Biomédica en Red en Enfermedades Neurodegenerativas-Instituto de Salud Carlos III (CIBER-CIBERNED-ISCIII), Madrid 28029, Spain; 4 Instituto Universitario de Investigación Biosanitaria de Extremadura (INUBE), Cáceres 10003, Spain.

**Keywords:** ER-mitochondria signalling, amyotrophic lateral sclerosis, frontotemporal dementia, C9orf72, dipeptide repeat polypeptides

ER-mitochondria contacts constitute key signalling hubs that coordinate complex intracellular processes, including calcium (Ca^2+^) homeostasis, lipid metabolism or mitochondrial energy production, among of others [1]. The specialized ER subdomains in contact with mitochondria are called mitochondria-associated membranes (MAMs). Vesicle-associated membrane protein-associated protein B (VAPB) is a MAM protein which interacts with protein tyrosine phosphatase-interacting protein 51 (PTPIP51), a protein of the outer mitochondrial membrane (OMM). The VAPB-PTPIP51 interaction acts as an inter-organelle bridge, tethering ER and mitochondria to facilitate ER-mitochondria signalling [1].

In neurons, ER and mitochondria are commonly found in synaptic terminals. The VAPB and PTPIP51 tethers localise and form contacts at synapses, which increase upon neuronal activity stimulation *in vitro*. Importantly, siRNA loss of VAPB or PTPIP51 perturbs synaptic function and dendritic spine morphology, highlighting the importance of ER-mitochondria communication and the VAPB-PTPIP51 interaction in the physiology and pathophysiology of neurons [2].

Indeed, alterations in ER-mitochondria signalling and the VAPB-PTPIP51 interaction have been related to age-related neurodegenerative diseases, such as Parkinson’s disease (PD), Alzheimer’s (AD) disease and Amyotrophic Lateral Sclerosis (ALS) and Frontotemporal dementia (FTD) [1].

ALS and FTD are considered to exist on a disease spectrum (ALS/FTD), which is clinically, genetically and pathologically related [3]. An hexanucleotide repeat expansion (HRE) in the non-coding region of the gene *C9orf72* is a leading cause of ALS/FTD) [3]. One of the proposed pathomechanisms underlying *C9orf72* HRE -mediated disease involves its unconventional translation into dipeptide repeat (DPR) polypeptides by repeat associated non-AUG (RAN) translation, generating five different DPRs (poly-GA, poly-GP, poly-GR, poly-PA and poly-PR) [3].

Our recent study, published in Aging Cell [4], explored the effect of *C9orf72* HRE on the VAPB-PTPIP51 tethers in rodent and cell culture models of *C9orf72*-linked ALS/FTD. To quantify the interaction between endogenous VAPB and PTPIP51 proteins *in situ* we performed proximity ligation assays (PLA). The PLA assays are suitable for quantifying inter-organelle communication contacts and PLAs, including ones for VAPB and PTPIP51, have already been used to quantify ER-mitochondria contacts [1].

First, we investigated the VAPB-PTPIP51 interaction in patient-derived induced pluripotent stem cell (iPSc) cortical neurons carrying the pathogenic *C9orf72* expansion. *C9orf72* HRE iPSc-neurons demonstrated reduced VAPB-PTPIP51 PLA signals compared to controls. A key function of the VAPB-PTPIP51 tethers is to facilitate ER-to-mitochondria Ca^2+^ delivery [5, 6] consistently, the functional connection between the ER-located inositol 1,4,5-triphosphate receptor (IP3R) type-1 Ca^2+^ channel and the OMM located voltage-dependent anion-selective channel-1 (VDAC1), was also reduced in these neurons.

The reduction in VAPB-PTPIP51 contact observed in iPSc-derived neurons was reproduced in an *in vivo* setting, a *C9orf72* transgenic mouse that carries a bacterial artificial chromosome (BAC) containing a human pathogenic 450 GGGGCC repeat expansion. These mice develop age-dependent accumulation of RNA foci and DPRs, a phenotype that is accompanied by loss of hippocampal neurons and impaired cognitive function at 12 months [7]. Early pathogenic changes are believed to be the most important; interestingly, the VAPB-PTPIP51 interaction was already reduced prior to disease onset, at 6 months of age in *C9orf72* BAC transgenics compared to non-transgenic controls. These findings indicated that changes in VAPB-PTPIP51 binding are an early feature of disease in this mouse model, supporting the notion that disruption of the VAPB-PTPIP51 tethers might contribute to disease.

As a major pathogenic mechanism for *C9orf72* HRE involves production of toxic DPR polypeptides poly-GA, poly-GR and poly-PR, we enquired whether these DPRs might disrupt VAPB-PTPIP51 and IP3R-VDAC1 interactions in cultured rat cortical neurons. For these experiments, we used DPRs plasmids that utilise alternative codon sequences that preclude formation of RNA foci. Expression of the toxic DPRs reduced both PLA signals and the co-localisation ER and mitochondrial markers assessed using super resolution structured illumination microscopy (SIM). When addressing ER-mitochondria Ca^2+^ transfer in a cell line commonly used as a neuronal model, we confirmed that pathogenic *C9orf72*-derived DPRs were also associated with a disruption to this major ER-mitochondria signalling function.

On a molecular level, our study placed activation of glycogen synthase kinase 3β (GSK-3β), a previously known inhibitor of ER-mitochondria communication, as the responsible for the DPRs-mediated effects on the VAPB-PTPIP51 interaction. GSK-3β is a ubiquitously expressed and constitutively active serine/threonine protein kinase involved in diverse physiological pathways ranging from metabolism, cell cycle to neuroprotection [1]. Across the literature, there is an interesting case to be made for GSK3 as a driver in the progression of chronic diseases related to age, including ALS/FTD. However, the mechanism by which GSK-3β is activated by *C9orf72*-derived DPRs or the precise mechanism by which GSK-3β might influence VAPB-PTPIP51 binding still remains unknown.

Damaged ER-mitochondria signalling is seen in other cell and transgenic mouse models of familial ALS/FTD, such as those involving TAR-DNA binding protein 43kDa (TARDBP), VAPB, Fused in Sarcoma (FUS), superoxide dismutase 1 (SOD1), sigma non-opioid intracellular receptor 1 (SIGMAR1) or TANK binding kinase 1 (TBK1) [1]. Where studied, this implicated the disruption of the VAPB-PTPIP51 tethers [1]. Importantly, the VAPB-PTPIP51 tethering interaction has been recently shown to be reduced in human sporadic ALS *post mortem* tissue as well [8].

There is an emergence of effective therapies for ALS/FTD patients. ER-mitochondria contacts sites pose a pivotal molecular target. Our study highlighted this emerging research avenue in drug discovery for these devastating diseases consolidating ER-mitochondria signalling as a common pathogenic mechanism, affecting to the most frequent familial form of ALS/FTD; pathogenic *C9orf72* repeat expansion account for around 40% and 25% of familial ALS or FTD respectively, and for around 6% of sporadic cases in both as well. Our work also determined that changes in this signalling are an early feature of disease in a *C9orf72*-mouse model, representing a key pathogenic event prior to the onset of symptoms. Furthermore, this work also revealed important new information about the role of DPRs species in *C9orf72*-mediated toxicity.

Although research in this area is currently very active, important gaps persist. For example, despite the implications of ER-mitochondria signalling contributing to age-related disorders, little is known about ER-mitochondria contacts regulation throughout an organism’s lifespan. In our study, we noticed age-related changes in ER-mitochondria contacts; we observed that in control non-transgenic mice, the numbers of VAPB-PTPIP51 PLA signals were fewer in 12-month compared to 6-month-old mice, while the molecular mechanisms underlying this age-dependent reduction are not clear at this stage.

The aging process in the brain associates with a loss of homeostasis and impaired energy metabolism, with mitochondrial dysfunction as a major hallmark of aging. Whether ER-mitochondria associations might drive a role in these age-related processes constitute an exciting new area of research.
